# Radiological aspects in computed tomography as determinants in the diagnosis of pulmonary tuberculosis in immunocompetent infants

**DOI:** 10.1590/0100-3984.2018.0025

**Published:** 2019

**Authors:** Teresa Cristina Sarmet dos Santos, Sérgio Setúbal, Alair Augusto Sarmet Moreira Damas dos Santos, Marcia Boechat, Claudete Aparecida Araújo Cardoso

**Affiliations:** 1 Universidade Federal Fluminense (UFF) - Hospital Universitário Antônio Pedro (HUAP), Niterói, RJ, Brazil.; 2 Instituto Nacional de Saúde da Mulher, da Criança e do Adolescente Fernandes Figueira (IFF/Fiocruz), Rio de Janeiro, RJ, Brazil.; 3 Universidade Federal Fluminense (UFF) - Faculdade de Medicina, Departamento Materno-Infantil, Niterói, RJ, Brazil.

**Keywords:** Tuberculosis, pulmonary, Children, Computed tomography, Tuberculose pulmonar, Crianças, Tomografia computadorizada

## Abstract

**Objective:**

To describe the chest computed tomography (CT) findings in immunocompetent children under 36 months of age with pulmonary tuberculosis.

**Materials and Methods:**

This was a descriptive case series conducted in the city of Rio de Janeiro, Brazil, between January 2004 and July 2013, involving 20 young children who underwent CT after undergoing chest X-rays that did not provide a definitive diagnosis.

**Results:**

All of the participants had lymph node enlargement and consolidations. In 15 cases (75%), the consolidations were accompanied by atelectasis. Pulmonary cavitation was seen in 10 cases (50%), and cavitation within consolidations was seen in 7 (35%). The areas of cavitation and parenchymal destruction were not seen on conventional chest X-rays.

**Conclusion:**

The radiological presentation of pulmonary tuberculosis in young children differs from that described in older children and adults. CT is an effective method for the early diagnosis of pulmonary tuberculosis in immunocompetent infants, allowing the rapid institution of specific treatment, which is crucial for halting disease progression, as well as for preventing local and systemic complications.

## Abstract (pt)

**Objetivo:**

Descrever achados radiológicos na tomografia computadorizada (TC) de tórax de crianças imunocompetentes menores de 36 meses com tuberculose pulmonar.

**Materiais e Métodos:**

Esta série de casos foi desenvolvida na cidade do Rio de Janeiro, no período de janeiro de 2004 a julho de 2013, onde 20 pacientes foram submetidos a TC após a realização de radiografias de tórax que não definiram o diagnóstico.

**Resultados:**

Todos os participantes tiveram linfonodomegalias e consolidações. Em 15 casos (75%) as consolidações tiveram atelectasia associada. Escavações pulmonares ocorreram em 10 casos (50%), havendo consolidações em 7 (35%). Áreas de escavação e destruição parenquimatosa em fase inicial não foram observadas nas radiografias simples.

**Conclusão:**

A apresentação radiológica de tuberculose pulmonar em lactentes não foi a mesma descrita em crianças maiores e adultos. A TC é um método aplicável para o diagnóstico precoce de tuberculose pulmonar em lactentes imunocompetentes, permitindo a rápida instituição de tratamento específico, que é crucial para interromper a progressão da doença e prevenir suas complicações locais e sistêmicas.

## INTRODUCTION

Tuberculosis continues to be a major global health problem. According to the World Health Organization, it is the leading infectious cause of death worldwide^(^^1^^)^. It is also a significant cause of morbidity and mortality in children living in tuberculosis-endemic areas^(^^2^^,^^3^^)^. In 2016 alone, approximately 10.4 million new cases of tuberculosis were diagnosed worldwide, and there were approximately 1.7 million deaths from the disease. In that same year, there were 66,796 new cases in Brazil, which now ranks 18th on the list of the 20 countries with the highest tuberculosis burden. Brazil accounts for 0.9% of the estimated cases worldwide and 33% of those in the Americas. In comparison with other Brazilian states, Rio de Janeiro has the second highest rate of tuberculosis incidence-61.2/100,000 population-and the highest rate of tuberculosis-related mortality-5.0/100,000 population^(^^4^^)^.

Tuberculosis is an infection of the airways. Most children acquire the infection at home after having been in contact with active tuberculosis cases (parents or caregivers). Adults with tuberculosis infect children in 30-40% of such cases, even when their infection is paucibacillary, with negative sputum culture results^(^^3^^)^. However, large pulmonary cavities are the most important factor determining how contagious the disease will be, because the high concentration of oxygen in such lesions favors an intense multiplication of bacilli, transforming tuberculosis patients into a significant source of environmental contamination^(^^1^^,^^5^^)^. The extent of lung involvement is another important factor for contagiousness, because the bacterial load, cough intensity/frequency, and the number of cavities can determine the propagation of the bacillus. In individuals with no prior contact with the bacillus, the primary infection can progress subclinically. When primary infection manifests as a disease, it is called primary tuberculosis^(^^1^^)^. Most cases of pulmonary tuberculosis in children are primary^(^^6^^-^^14^^)^.

Given the difficulty in detecting bacilli in clinical specimens collected from children, the diagnosis of childhood tuberculosis is often based on the tuberculin skin test results, as well as on epidemiological, clinical, and radiological evidence^(^^3^^)^. Diagnosing tuberculosis in children poses a major challenge. Many pediatric patients (65-95%) present with nonspecific clinical findings or a nonproductive cough. Therefore, the diagnosis of tuberculosis in this age group depends on a thorough anamnesis, a complete screening of contacts, and radiological imaging^(^^3^^,^^6^^,^^9^^,^^12^^)^. The World Health Organization recommends that tuberculosis be diagnosed on the basis of contact tracing and chest X-rays, even in the absence of a positive tuberculin skin test. However, chest X-rays are not a good indicator of tuberculosis in children, because they have a sensitivity of only 40%^(^^8^^)^. That could have negative consequences for children with a history of contact with tuberculosis, in whom normal chest X-ray findings might lead to diagnosis of latent tuberculosis infection instead pulmonary tuberculosis, and hence undertreatment only with isoniazid instead a complete treatment with four drugs.

Computed tomography (CT) is superior to conventional chest X-ray and can detect changes in children whose chest X-rays are normal or inconclusive^(^^8^^)^. In comparison with conventional chest X-rays, CT scans are much more sensitive in detecting the cavitations that can occur in children^(^^5^^,^^6^^)^. CT is also the method of choice to detect mediastinal and perihilar lymph nodes, which are very common in childhood tuberculosis, even if the lymph nodes are small^(^^11^^)^. CT shows lymph node enlargement in 60% of tuberculosis patients whose chest X-rays are normal^(^^8^^)^. In addition to mediastinal abnormalities, high-resolution CT scans can reveal findings in the lung parenchyma-such as miliary and centrilobular nodules-in patients with no evidence of such lesions on conventional chest X-rays. Studies on chest X-ray findings of tuberculosis in children under 36 months of age are relatively scarce in the literature, and such studies typically employ conventional chest X-rays^(^^4^^,^^6^^,^^8^^,^^11^^-^^26^^)^.

We were motivated to conduct this study because we were made aware of CT findings of pulmonary cavitation in infants with tuberculosis in the radiology departments of two different hospitals in the state of Rio de Janeiro, Brazil. The main objective of the study was to describe the radiological findings of tuberculosis in children under 36 months of age. We present a detailed definition of the most common high-resolution CT findings in such patients, with the aim of helping reduce errors and delays in the diagnosis of tuberculosis among immunocompetent patients in this age group.

## MATERIALS AND METHODS

This was a study of 20 consecutive cases of tuberculosis in immunocompetent children 1-36 months of age seen at two public hospitals in the state of Rio de Janeiro, Brazil between January 2004 and July 2013. The inclusion criteria were as follows: having undergone a chest X-ray and a CT scan at both hospitals; being ≤ 36 months of age; and having been diagnosed with pulmonary tuberculosis, as per the below-mentioned criteria. Children with any form of immunosuppression (HIV/AIDS, lymphoproliferative disease, or immunosuppressive drug therapy) were excluded from the study. The study was approved by the human research ethics committees of both institutions, in compliance with the ethical standards currently in force.

A diagnosis of tuberculosis was established when two or more of the following criteria were met: positive bronchoalveolar or gastric lavage culture; previous contact with an adult with active tuberculosis; positive tuberculin skin test; and regression of radiological and clinical signs after the institution of specific treatment. We could not find any other possible causes for the clinical and radiological findings in any of the patients. All of the patients presented complete remission of symptoms and radiological improvement after the initiation of treatment for pulmonary tuberculosis.

Nineteen patients received the bacillus Calmette-Guérin vaccine. All of the patients were submitted to a tuberculin skin test, and 13 (65%) of them had a positive result. Ten patients (50%) had a history of contact with individuals with active pulmonary tuberculosis.

All patients underwent conventional posteroanterior chest X-rays as part of the initial assessment and were under radiographic follow-up for a mean period of two years. CT scans were performed 1-10 days after the chest X-ray (mean: 6 days). The indications for CT included the following: to investigate unusual findings on chest X-rays, such as mass-like pseudotumors and diffusely distributed nodules; to identify or confirm lymph node enlargement; to detect or evaluate complications, such as narrowing of the upper airways, atelectasis, emphysema, and pleural or pericardial tuberculosis; and to clarify diagnoses of cystic adenomatoid malformation, mediastinal tumor, or infectious neurological complications.

All chest CT scans were obtained through volumetric acquisition with 5-mm collimation, after intravenous administration of iodinated contrast for the assessment of the mediastinum, with a slice thickness of 0.6-1 mm, an interslice gap of 8-12 mm, and a high-resolution technique to assess the lung parenchyma. Following the "as low as reasonably achievable" principle, which refers to the mandatory principle of keeping radiation doses applied to patients and technical staff as low as reasonably possible, the peak kilovoltage (kVp) and milliamperage (mAs) parameters were adjusted to 120-100 kVp and 30-100 mAs, respectively^(^^27^^)^. When necessary, sedation with a single dose of 80 mg/kg of 16% oral chloral hydrate was used, and there were no sedation-related complications.

All chest X-rays and CT scans were reviewed. We then described the radiological findings, according to their type, number, location, and characteristics, using the terminology found in the radiology literature of Brazil^(^^28^^)^. The type and frequency of local and systemic complications were also registered. Two radiologists, working independently, analyzed all of the images, and any disagreements were resolved by consensus. The following data were evaluated: age and gender; multiple simultaneous radiological findings; consolidation/atelectasis; cavitation; nodules (size and type); ground-glass opacities; hilar or mediastinal lymph node enlargement, with or without calcifications; airspace disease; signs of upper airway obstruction; local complications; and systemic complications.

## RESULTS

Twenty immunocompetent infants with pulmonary tuberculosis-12 boys and 8 girls, with a mean age of 18 months (range, 1-36 months)-were evaluated during the study period (Table 1). In all 20 cases, the radiological findings were multiple and varied, being bilateral in 90%.

**Table 1:** CT findings in immunocompetent infants with pulmonary tuberculosis.

Case no.	Age (months)	Gender	Multiple findings	Bilateral findings	Consolidation/ atelectasis (n)	Cavitation (n)	Nodules	Type of nodules	Ground-glass	Lymph node enlargement (n)	Type of lymph node enlargement	Air trapping	Airway obstruction
1	8	F	Yes	Yes	3	3	Yes	Centrilobular	Yes	5	Hypodense center	Yes	Yes
2	3	F	Yes	Yes	2	2	Yes	Centrilobular	Yes	5	Hypodense center	Yes	Yes
3	12	M	Yes	Yes	1	-	-	-	-	2	Calcifications	Yes	Yes
4	12	M	Yes	Yes	2	-	-	-	-	3	Hypodense center	-	Yes
5	3	M	Yes	Yes	2	1	-	-	Yes	6	Hypodense center	Yes	-
6	5	M	Yes	Yes	6	1	Yes	Centrilobular	-	4	Calcifications	Yes	Yes
7	5	M	Yes	Yes	3	3	Yes	Miliary	-	6	Hypodense center	Yes	-
8	24	M	Yes	Yes	2	-	-	-	-	5	Hypodense center	-	-
9	6	F	Yes	Yes	1	1	Yes	Centrilobular	-	3	Calcifications	Yes	-
10	8	F	Yes	Yes	3	-	-	-	Yes	6	Hypodense center	Yes	Yes
11	21	F	Yes	Yes	2	2	Yes	Centrilobular	Yes	6	Calcifications	Yes	-
12	28	M	Yes	Yes	3	-	Yes	Centrilobular	Yes	2	Hypodense center	-	Yes
13	36	M	Yes	Yes	2	-	Yes	-	Yes	4	Hypodense center	Yes	Yes
14	3	F	Yes	Yes	4	2	-	-	Yes	6	Hypodense center	Yes	Yes
15	1	M	Yes	Yes	3	2	Yes	Centrilobular	Yes	4	Hypodense center	Yes	Yes
16	5	M	Yes	Yes	3	-	Yes	Centrilobular	-	2	Hypodense center	-	-
17	7	F	Yes	No	2	1	-	-	-	2	Hypodense center	Yes	Yes
18	12	M	Yes	Yes	1	-	-	-	-	4	Calcifications	Yes	Yes
19	22	F	Yes	Yes	2	2	-	-	-	3	Calcifications	Yes	Yes
20	5	M	Sim	No	2	2	-	-	-	4	Hypodense center	Yes	Yes

F, female; M, male.

**Figure 1:**
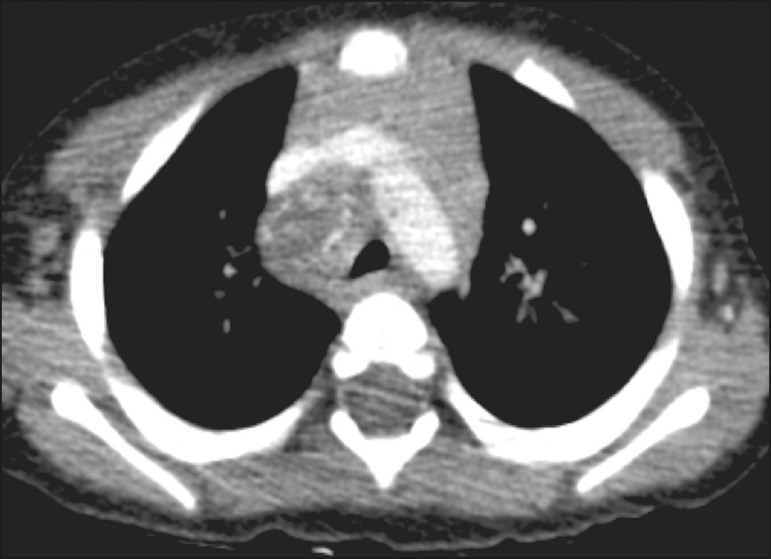
Contrast-enhanced CT, with soft-tissue window settings, of na eight-month-old infant, showing right paratracheal retrocaval lymph node enlargement with a hypodense core and peripheral contrast enhancement, suggestive of central necrosis.

**Figure 2:**
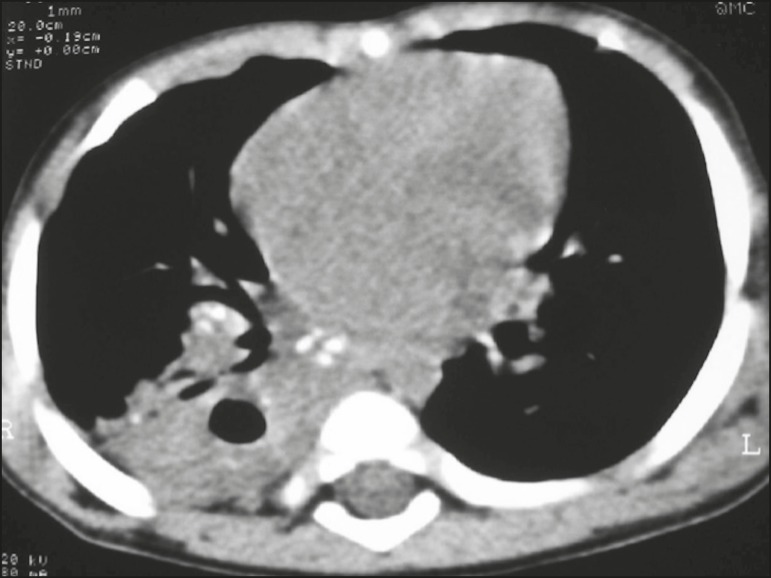
Noncontrast CT, with soft-tissue window settings, of a five-monthold infant, showing consolidation, containing air bronchograms and cavitation, in the posterior basal segment of the right lower lobe. Note the enlarged subcarinal lymph nodes with calcifications.

The main radiological finding was lymph node enlargement (Figure 1), which was seen in all of the patients. The enlarged lymph nodes contained calcifications (Figure 2) in eight patients (40%) and caused direct bronchial compression in eight (40%). The frequency of right paratracheal lymph node involvement was 95%. The enlarged nodes were pretracheal in 15 patients (75%), and subcarinal in 13 (65%). In 14 patients (70%), the enlarged nodes showed a hypodense core after contrast infusion (Figure 1), suggesting severe edema or necrosis.

**Figure 3:**
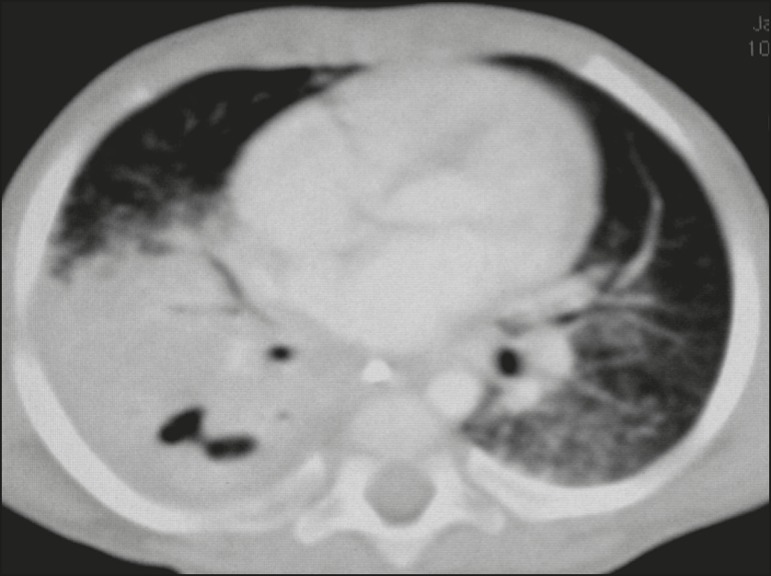
CT, with lung parenchymal window settings, showing a consolidation, with an atelectatic component and an area of cavitation, in the right lower lobe. A ground-glass opacity can be seen in the left lower lobe.

**Figure 4:**
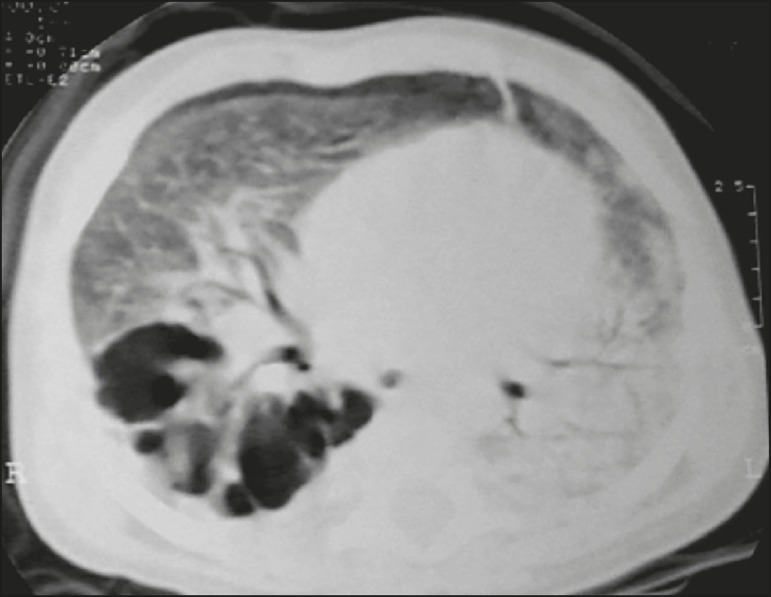
CT, with lung parenchymal window settings, of a three-month-old infant, showing bullous lesions with expansile features in the right lower lobe and consolidation with an atelectatic component in the left lower lobe. Note the respiratory motion artifacts in the retrosternal region.

**Figure 5:**
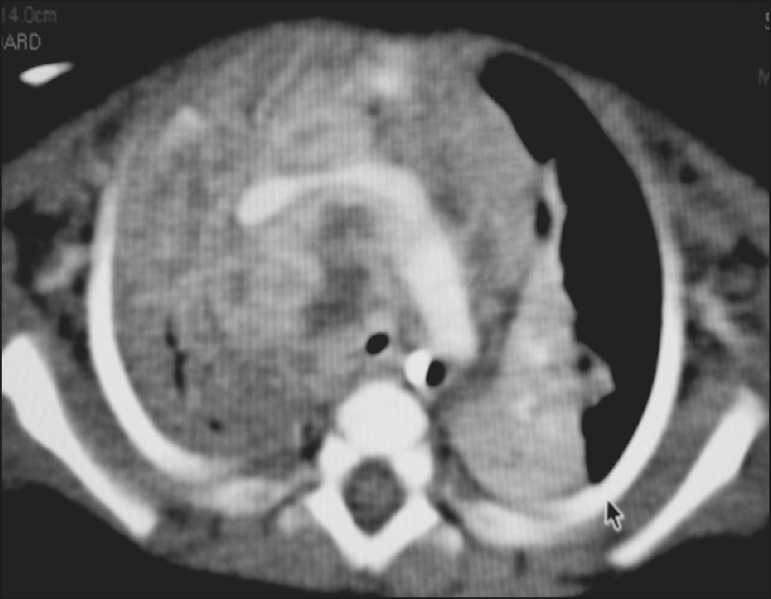
Pulmonary tuberculosis presenting as a pseudotumor in a threemonth-old infant. Contrast-enhanced CT, with soft-tissue window settings, showing enlarged hypodense lymph nodes-pretracheal, right paratracheal, and caval-aortic-with expansile features, forming a mass that partially compressed the right primary bronchus. Note the consolidations in the upper lobes and in the apical segment of the left lower lobe.

**Figure 6:**
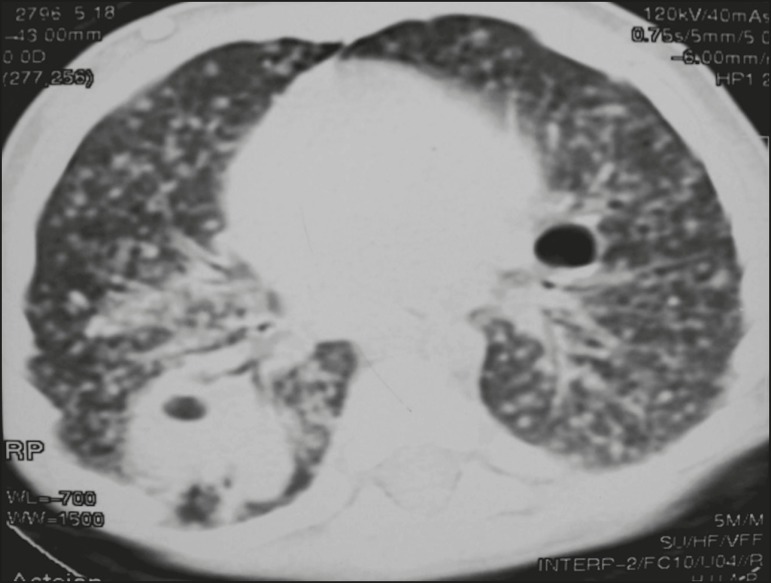
CT, with lung parenchymal window settings, of a five-month-old infant, showing randomly distributed (miliary) nodules in both lungs. Note the area of consolidation containing cavitation in the posterior basal segment of the right lower lobe. Another thin-walled cavitation can be seen in the lingula.

**Figure 7:**
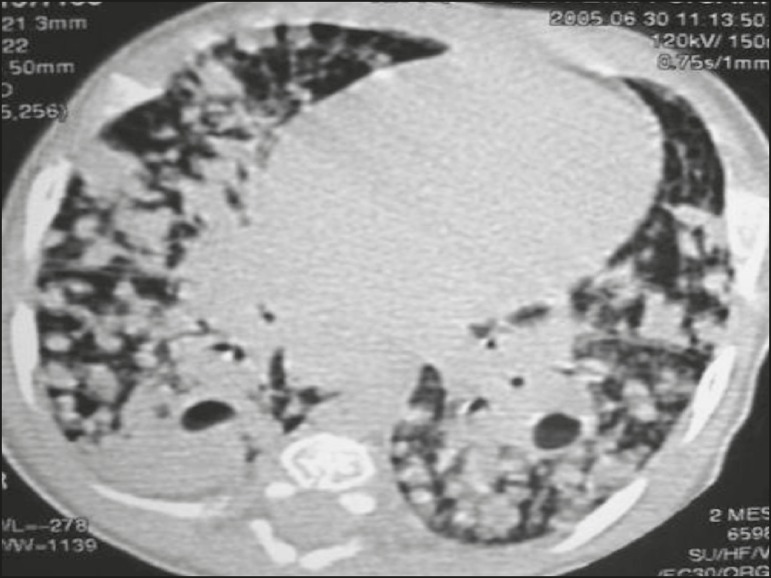
CT, with lung parenchymal window settings, of a two-month-old infant, showing airspace nodules, ranging in size from 5 mm to 10 mm, diffusely distributed in both lungs, together with consolidations containing cavitations in the lower lobes.

Airspace consolidations, with or without atelectasis, were found in all 20 patients (Figures 3 and 4). A mass-like consolidation (pseudotumor) was found in one case only (Figure 5). Cavitations were seen in 10 (50%) of the patients (Figures 2, 3, 4, 6, and 7), although they differed in some respects. A six-month-old infant presented cavitation and retractile opacities, accompanied by signs of chronic lung disease (honeycombing). All parenchymal cavitations were accompanied by radiological findings consistent with pulmonary consolidation and bronchial dissemination (airspace nodules, centrilobular nodules, and a tree-in-bud pattern), which most likely progressed from a primary focus, known as the Ghon complex. One patient had cavitations that evolved to extensive bullous lobar lesions (Figure 4) and developed a systemic complication (meningoencephalitis). Nine patients (45%) had disseminated pulmonary nodules. In four of those patients (20%), the nodules were between 5 mm and 10 mm in diameter (Figure 7), whereas in the other five (25%), they were between 2 mm and 4 mm in diameter. Five of those nine patients also presented ground-glass attenuation. Only one patient presented miliary nodules (Figure 6), which were suggestive of direct hematogenous dissemination with concomitant involvement of the central nervous system.

Air trapping occurred in 16 patients (80%), usually with a segmental or lobar distribution. In five patients (25%), more than one lobe was involved.

Other local findings and complications were bronchiectasis, bronchiolectasis, pleural thickening, pleural effusion, pneumothorax (in four patients), bullae (in four patients), and esophageal perforation (in two patients). The most common systemic complication was meningoencephalitis (in four patients). In 19 patients, the tuberculosis treatment was initiated immediately after CT. Only one patient received the radiological diagnosis after initiation of the drug therapy.

## DISCUSSION

Children with tuberculosis present pathophysiological and immunological responses that differ from those seen in adults^(^^8^^)^. Most cases of childhood tuberculosis are primary, typically presenting enlarged lymph nodes and no cavitation. However, in some cases of primary tuberculosis in children, cavitations can develop, which might lead to confusion with post-primary, reactivation, or adult-type tuberculosis. Therefore, the only accurate predictor of this X-ray finding is the immune status of the patient, rather than the time since tuberculosis acquisition^(^^9^^)^. Knowledge about the pathophysiological progression of the disease is essential for understanding the pathogenesis of the chest X-ray findings^(^^5^^)^.

Imaging tests have recently been the focus of studies in the radiology literature^(^^29^^-^^35^^)^. Although there have been several studies on the radiological imaging of childhood tuberculosis, our study is comparable only to those that have used CT and have involved patients in the same age group. Among such studies, those conducted by Delacourt et al.^(^^15^^)^, Kim et al.^(^^6^^,^^14^^)^, Pereira et al.^(^^9^^)^, and Peng et al.^(^^7^^)^ are the most similar to ours. However, Pereira et al.^(^^9^^)^ analyzed only four cases and Peng et al.^(^^7^^)^ attempted to establish imaging differences between tuberculosis and community-acquired pneumonia. Our radiological findings were similar to the findings reported in those studies, except for the greater number of cases with cavitary lesions in our study.

Lymph node enlargement is quite common in childhood tuberculosis and often manifests as extrinsic airway compression, which is a well-known complication of lymphadenopathy in primary tuberculosis. When the airway is partially obstructed, a ball-valve effect can occur, leading to distal hyperinflation; whereas when the obstruction is complete, the distal air is reabsorbed, leading to collapse of the lung parenchyma, with or without necrosis^(^^5^^)^. In tuberculosis, calcified lymph nodes and lymph nodes with central necrosis are common, although such lymph nodes are difficult to see on conventional chest X-rays^(^^7^^)^. In addition, conventional chest X-rays lack sensitivity to detect lymph node enlargement, which is the most important and common finding in childhood tuberculosis^(^^10^^)^. In the present study, lymph node enlargement was seen in 100% of the cases and calcified lymph nodes were observed in 35%. In 40% of the cases, lymphadenopathy caused bronchial compression. In 95%, the lymph nodes presented central necrosis. Obstruction of the upper airways by direct or indirect bronchial compression was another frequent finding (seen in 70% of the cases). The frequency of lymph node enlargement and its complications in the present study is in agreement with the findings of the aforementioned studies and underscores the importance of using CT in this clinical context^(^^6^^-^^10^^)^.

Various studies have shown that consolidations are the most common imaging findings in childhood tuberculosis^(^^3^^,^^5^^,^^6^^,^^8^^,^^9^^,^^11^^)^. Airway involvement due to lymphadenopathy can lead to dissemination of the disease and the development of bronchopulmonary consolidations. The studies cited above have indicated the importance of persistent parenchymal opacities that do not improve after the use of antibiotics in this age group. Other studies^(^^8^^,^^21^^,^^36^^)^ have reported mass-like pseudotumors-without satellite lymph node enlargement-in children under two years of age, although this is considered an uncommon finding. All of the patients in our sample presented consolidations. The radiological pattern of lobar consolidation was seen in 15 cases (75%). Consolidations with the appearance of a pseudotumor, resulting in a mass effect, were seen in one case.

In the present study, parenchymal calcifications were seen in only two of the cases, both of them prior to the institution of specific treatment. Parenchymal calcifications were also seen in three of the four cases evaluated by Pereira et al.^(^^9^^)^, also before the specific treatment was initiated. In most studies^(^^5^^,^^10^^,^^21^^)^, calcifications were seen only after ≥ 10 months of follow-up. Marais et al.^(^^2^^)^ stated that calcifications in preschool children are usually seen after a relatively short period of time. Those authors believe that their occurrence might be related to the immune response, disease progression, and initiation of specific treatment. They also believe that this finding could therefore be used as an additional diagnostic criterion for tuberculosis. In our sample, lymph node and parenchymal calcifications occurred in 40% of the patients and were probably related to an immune response evoked by long-standing disease, and calcification could therefore be considered another diagnostic criteria. When such calcifications are accompanied by lymphadenopathy, central necrosis, and signs of bronchogenic dissemination, a diagnosis of tuberculosis becomes more probable.

It is common for cavitations to appear within consolidations. Chest CT usually shows areas of cavitation and parenchymal destruction that are not seen on conventional chest X-rays^(^^4^^)^. Kim et al.^(^^6^^)^, for instance, found cavitations within parenchymal lesions on the chest X-rays in two (8%) of 25 patients evaluated. However, 17 of those patients subsequently underwent CT, which revealed multifocal areas of low attenuation within consolidations in seven (41%) and well-defined cavitations in five (29%). The cavitation evolved to extensive bilateral bullous lesions in one patient, who subsequently died^(^^6^^)^.

Cavitations are considered a classical manifestation of post-primary or adult-type tuberculosis. However, there are two other possible mechanisms involved in the formation of cavitations in children^(^^4^^)^: gradual dissemination from the Ghon complex; and bronchial obstruction by lymph nodes. Griffith-Richards et al.^(^^5^^)^ evaluated children with pulmonary tuberculosis and identified cavitations in 63%. In the present study, we identified cavitations in ten (50%) of the 20 cases evaluated and cavitations within a consolidation in seven (35%). One 3-month-old infant presented extensive bullous lesions, initially diagnosed as a cystic adenomatoid malformation.

Miliary tuberculosis, one of the most severe forms of tuberculosis, is secondary to the hematogenous dissemination of bacilli. Tuberculosis can progress to miliary tuberculosis at any point, such progression being due to the inability of the organism to control the infection. Miliary nodules can therefore coexist with elements of the primary complex, larger opacities, or cavitations^(^^24^^)^. Pulmonary tuberculosis can be fatal if it is not diagnosed and treated early. Diagnosing it can be difficult, given that the initial symptoms are nonspecific and the typical chest X-ray findings appear relatively late^(^^37^^)^. Miliary tuberculosis is an interstitial disease, presenting clinical, radiological, and physiological similarities with other diseases of its type, which makes early diagnosis even more problematic. That is of great importance because miliary tuberculosis is treatable, whereas many other interstitial lung diseases are either untreatable or are much more difficult to treat^(^^38^^)^.

Local and systemic complications of pulmonary tuberculosis, observed in 40% and 25% of the patients in our sample, respectively, are indicators of the potential severity of the disease. In this context, CT is an invaluable method. Radiologists and pediatricians must be aware of the importance of these findings in an immunocompetent infant and must always correlate them with the family history. Early diagnosis and timely initiation of the appropriate treatment are crucial to halting tuberculosis progression and preventing its local and systemic complications, which might be irreversible.
